# Case-report: A rare cause of an intra-abdominal mass^☆^

**DOI:** 10.1016/j.ijscr.2019.10.080

**Published:** 2019-11-12

**Authors:** Amanda Shabana, Farzan Dholoo, Rebecca Nunn, Waseem Hameed

**Affiliations:** a Wexham Park Hospital, Frimley Health NHS Foundation Trust, General Surgery, Berkshire, UK; b Frimley Park Hospital, Frimley Health NHS Foundation Trust, General Surgery, Surrey, UK

**Keywords:** Abdominal, mass, Abdominal, pain, Cyst, Mesenteric, cyst, ’s and, surgical, techniques

## Abstract

**Introduction:**

Mesenteric cysts are a rare and often asymptomatic incidental finding on imaging. The diagnosis and management of mesenteric cysts remains a clinical challenge since those presenting with symptoms, often have vague and nonspecific symptoms, owing to variability in cyst location and size. This case report will aim to discuss the presentation, investigation and management options available.

**Presentation:**

A 73-year-old female presented with abdominal swelling and a palpable right sided mass. Examination revealed a right sided mass and abdominal distention with vital signs within normal limits. Ultrasound scan (USS) revealed a right-sided 12 cm × 11 × cm × 8 cm thin walled cyst.

**Discussion:**

Mesenteric cysts are mostly asymptomatic but can cause nonspecific symptoms of abdominal pain, abdominal distension, altered bowel habit, nausea, vomiting and an abdominal mass. Complete surgical excision, either laparoscopically, or through a laparotomy is typically considered first line treatment. If size or location of the cyst precludes complete surgical excision, partial excision with marsupialisation of the opening of the cyst into the abdominal peritoneal cavity is a second option.

**Conclusion:**

Mesenteric cysts represent a rare cause of intra-abdominal mass. Owing to low prevalence, literature is limited as is guidance on management. Careful pre-operative planning is essential so as to avoid operative complications. Imaging such as USS is of great importance; however Computed Tomography (CT) and Magnetic Resonance Imaging (MRI) may be of more benefit, owing to the complex anatomical relations within which mesenteric cysts can present. Surgical excision is widely reported as the surgical treatment of choice for symptomatic cysts.

## Highlights

• Mesenteric cysts may present with symptoms of early saiety and bloating.
• Mesenteric cyst anatomy may be difficult to delineate on Ultrasound imaging alone.
• Mesenteric cyst drainage and marsupialisation is a safe alternative to excision.

## 1 Introduction

Mesenteric cysts are rare and occur at any age, with an incidence of 1 in 250,000 hospital admissions [2]. Their existence was first described by Benivieni, an Italian anatomist, in 1507 during an autopsy [3]. Mesenteric cysts are mostly located in the ileum (60 %), ascending colon (24 %), retroperitoneum (14.5 %) and the omentum [4]. Clinical presentation is variable: often they are asymptomatic and found incidentally when patients are receiving work up for other conditions [5]. When patients are symptomatic, the most common presenting complaints are: pain (82 %), nausea and vomiting (45 %), constipation (27 %), and diarrhoea (6 %). An abdominal mass is present in up to 61 % of patients [6]. Mesenteric cysts are usually benign with malignant cysts occurring in less than 3 % of cases [3]. Multiple studies highlight ultrasound as a suitable investigation, in the initial assessment of a potential abdominal mass, as the results are high yielding and usually highlight well defined cystic masses [7]. CT and MRI can help visualise fluid attenuation of potential lesions, as well as relationships to any nearby anatomy [8]. Complete surgical removal extending to excision of neighbouring viscera is considered first line in symptomatic cysts. Complete excision (extending to nearby viscera) is of paramount importance in preventing malignant transformation or other associated complications [9]. Furthermore complete surgical excision is associated with a lower recurrence rate as opposed to alternative therapies [10].

## 2 PRESENTATION OF CASE

A 73-year-old female presented to her General Practitioner (GP) with a history of abdominal swelling and a right sided abdominal mass. She also complained of early satiety and intermittent bloating but was otherwise systemically well with no fever. Notably absent were symptoms of: abdominal pain, vomiting, urinary symptoms, change in bowel habit or significant weight loss. Her past medical history included hypertension and atrial fibrillation, for which she was taking regular antihypertensive medications and warfarin. Previous surgeries included a hysterectomy and appendicectomy. She was fully independent, an ex-smoker, with no significant alcohol history. There was no family history of relevance or any congenital abnormality. On clinical examination, vital parameters were within normal limits with no lymphadenopathy, jaundice, pallor or pedal oedema present. On abdomen examination, her abdomen was distended but soft, with no obvious shifting dullness. A well-defined right sided intraabdominal mass could be felt on palpation, which was non-tender, with well-defined margins and cystic in consistency.

**Fig. 1:**
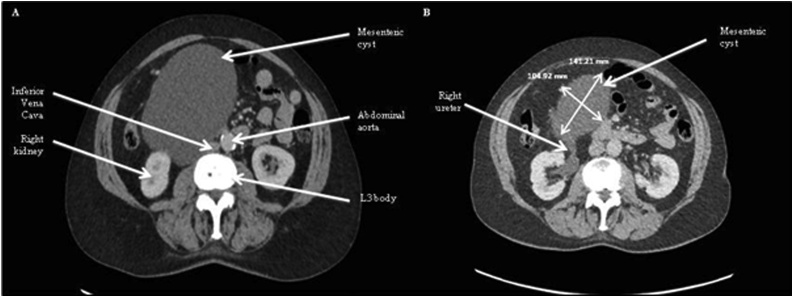
CT Abdomen and Pelvis demonstrating the anatomical relations of the mesenteric cyst (A). Note the relationship between the mesenteric cyst and the right ureter (B). Measurements of the cyst can also be found (B) although not to scale.

Blood tests including full blood count, urea and electrolytes, c-reactive protein and liver function tests were conducted which were unremarkable and within normal limits. Tumour markers were not requested. In the first instance an ultrasound of the abdomen was arranged which revealed a right-sided 12 cm × 11 × cm × 8 cm thin walled intrabdominal cystic mass. The origin of the cyst was difficult to determaine but USS suggest the mass may reporesent an exophytic right renal cyst. A follow up CT scan was requested, with the patient being referred to Urology multi disciplinary team (MDT) clinic. Once completed the CT scan report demonstrated that the cystic lesion in the abdomen on the right side was abutting the right kidney but did not appear to be arising from it. The organ of origin was still uncertain. There were no radiological features suggestive of malignancy, and no significant abnormalities of the solid abdominal or pelvic viscera (Fig. 1).

Following the CT scan findings, which suggested that the cyst was separate from the right kidney, the case was referred on to the general surgery team. MDT discussions concluded that this large cystic mass was likely to represent a ‘mesenteric cyst’ and operative management was recommended. The differential diagnosis of a mesenteric cyst includes: pancreatic pseudocysts, omental cyst, ovarian cyst, haemangiomas, endometriosis, loculated ascites (usually caused by tuberculous meningitis), mesothelioma and lymphoma [11].

**Fig. 2:**
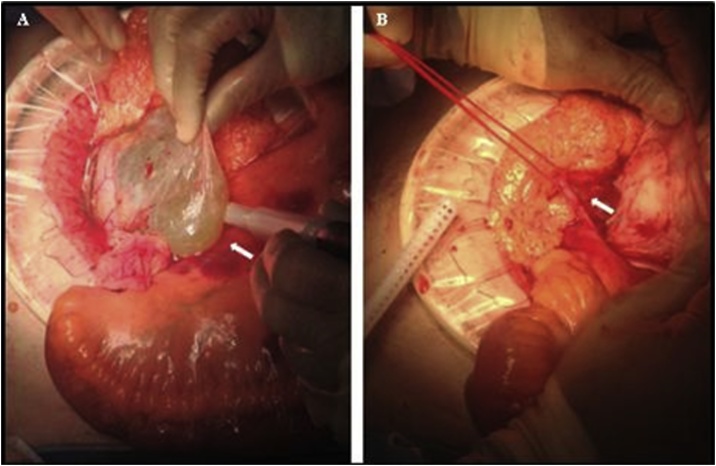
Images taken intra-operatively during the removal of the mesenteric cyst (A, See white arrow). Extreme care had to be taken during the procedure owing to the relationship between the mesenteric cyst and the right ureter (B, See white arrow).

Surgical management was deemed the most appropriate intervention starting with a laparoscopic approach, which showed that the right ureter appeared adherent to the anterior cyst wall, a relationship not previously identified on imaging. Given this anatomical relationship the surgery was converted to an open laparotomy. A mesenteric window was opened to access the cyst, and the ureter was preserved (Fig. 2). Due to complex anatomical relationships, and unfortunate intra-operative cyst rupture, it was deemed safer to drain and marsupialise the cyst, rather than pursuing complete excision. The patient recovered well post operatively and was discharged home after an uneventful 4-day post-operative inpatient stay. Histology of the cyst revealed a benign mesenteric cyst, likely lymphatic in origin. At a post-operative follow up period of 2 months, there were no significant symptoms and no clinical evidence of recurrence.

## 3 Discussion

Mesenteric cysts are rare intra-abdominal lesions located anywhere in the mesentery of the gastrointestinal tract from duodenum to rectum. The incidence is 1 in 250,000 hospital admissions [1]. They can occur in both large and small bowel mesentery, most commonly arising from ileal small bowel mesentery [3]. They were first described by Benivieni, at autopsy of an eight-year-old boy in 1507, while Tillaux performed the first successful surgery of a mesenteric cyst in 1880 [3]. Literature varies with regards to the aetiology of mesenteric cysts, a widely accepted theory however revolves around them originating from benign proliferations of mesenteric lymphatic tissue that fails to associate with the lymphatic system [12]. Mesenteric cysts can occur at any age. They are often asymptomatic and found incidentally when patients are undergoing investigations or treatment of another condition [4]. Clinical presentation includes nonspecific abdominal pain, abdominal distention, change in bowel habit, nausea, vomiting and an abdominal mass [5]. Rarely, they may present as an acute abdomen as a result of a complication such as intestinal obstruction, ischaemic bowel, volvulus and peritonitis or haemorrhagic shock secondary to rupture or bleeding into the cyst. Mesenteric cysts are typically benign; and histopathological classification has been described using six groups: lymphatic, mesothelial, enteric, urogenital, dermoid and pseudocysts [13]. Although mesenteric cysts are usually benign, the differentials of which they can mimic are usually serious life threatening conditions; therefore until a confirmed diagnosis is reached, an extensive history, examination and further investigations (including imaging) is of paramount importance. Abdominal USS can be useful in initial evaluation of an abdominal mass and can show fluid levels due to chyle and lymph [8]. CT scan and/or MRI scan may demonstrate the relationship between the cyst and nearby structures and blood vessels. In the absence of any robust randomized controlled clinical trials (at the time of publication), complete surgical excision by laparoscopic or an open surgical approach involving separating the cyst from the surrounding leaves of mesentery, is widely reported as the surgical treatment of choice for symptomatic cysts; with a lower recurrence rate compared to simple drainage or marsupialization [9]. It is of course important to note that conservative cyst management always remains a potential treatment route; however there again remains a significant lack of literature and trials in regards to this.

## 4 Conclusion

Mesenteric cysts are a rare cause of an intra-abdominal mass. They are found in 1 in 250 000 hospital admissions and represent an uncommon cause of abdominal mass. The aetiology is unclear but one suggested theory is that the mesenteric lymphatic tissue proliferates and fails to communicate with the core lymphatic system. Although mesenteric cysts are mostly asymptomatic they can cause nonspecific symptoms of abdominal pain, abdominal distention, altered bowel habit, nausea, vomiting and an abdominal mass. A careful history, examination and investigation are essential for both the diagnoses and management of these cases. The recommended management option of choice is complete surgical excision either laparoscopically or through a laparotomy. If this is not an option due to the size of the cyst or location; partial excision with marsupialisation of the opening of the cyst into the abdominal peritoneal cavity is a second option.

We do however note the rarity in incidence, of mesenteric cysts. There remains a significant clinical need for trials to help determine optimum management, surgical approaches and indeed potential novel surgical techniques for management.

## Declaration of Competing Interest

Farzan Dholoo – Nothing to declare.

Amanda Shabana - Nothing to declare.

Rebecca Nunn – Nothing to declare.

Waseem Hameed – Nothing to declare.

## Funding

Farzan Dholoo – Nothing to declare.

Amanda Shabana - Nothing to declare.

Rebecca Nunn – Nothing to declare.

Waseem Hameed – Nothing to declare.

## Ethical approval

The following statement applies for all listed authors:

Appropriate consents, permissions and releases have been obtained from the patient – We have a copy of the signed consent form.

## Consent

The following statement applies for all listed authors:

Written informed consent was obtained from the patient for publication of this case report and accompanying images. A copy of the written consent is available for review by the Editor-in-Chief of this journal on request”.

We do not have ethics committee approval as this was not a study on multiple patients.

No identifying details are included.

## Author contribution

Amanda Shabana and Farzan Dholoo are primary authors.

Rebecca Nunn is a second author.

Waseem Hameed is the responsible Consultant Surgeon, who: oversaw care of the patient; oversaw the project; edited the manuscript up to submission and supervised/facilitated the project up to submission.

## Registration of research studies

The following statement applies for all listed authors:

There was no research involving human participants.

There was no trials or observational research undertaken.

This is a case report only.

## Guarantor

Mr Waseem Hameed (General Surgery Consultant).

Waseem Hameed, Wexham Park Hospital, Frimley Health NHS Foundation Trust, General Surgery, Berkshire UK.

## Provenance and peer review

Not commissioned, externally peer-reviewed.
